# Deregulation of HMGA1 expression induces chromosome instability through regulation of spindle assembly checkpoint genes

**DOI:** 10.18632/oncotarget.3944

**Published:** 2015-05-15

**Authors:** Giovanna Maria Pierantoni, Andrea Conte, Cinzia Rinaldo, Mara Tornincasa, Raffaele Gerlini, Antonella Federico, Davide Valente, Enzo Medico, Alfredo Fusco

**Affiliations:** ^1^ Istituto di Endocrinologia ed Oncologia Sperimentale del CNR and Dipartimento di Medicina Molecolare e Biotecnologie Mediche, Università di Napoli “Federico II”, 80131, Naples, Italy; ^2^ Istituto di Biologia e Patologie Molecolari del CNR c/o Università “Sapienza” di Roma, 00185, Rome, Italy; ^3^ Laboratorio di Oncogenomica, Istituto per la ricerca sul cancro, 10060 Candiolo, Turin, Italy

**Keywords:** HMGA1, CIN, SAC, transcriptional regulation

## Abstract

The mitotic spindle assembly checkpoint (SAC) is an essential control system of the cell cycle that contributes to mantain the genomic stability of eukaryotic cells. SAC genes expression is often deregulated in cancer cells, leading to checkpoint impairment and chromosome instability. The mechanisms responsible for the transcriptional regulation and deregulation of these genes are still largely unknown. Herein we identify the nonhistone architectural nuclear proteins High Mobility Group A1 (HMGA1), whose overexpression is a feature of several human malignancies and has a key role in cancer progression, as transcriptional regulators of SAC genes expression. In particular, we show that HMGA1 proteins are able to increase the expression of the SAC genes *Ttk, Mad2l1, Bub1* and *Bub1b*, binding to their promoter regions. Consistently, HMGA1-depletion induces SAC genes downregulation associated to several mitotic defects. In particular, we observed a high number of unaligned chromosomes in metaphase, a reduction of prometaphase time, a delay of anaphase, a higher cytokinesis time and a higher percentage of cytokinesis failure by using live-cell microscopy. Finally, a significant direct correlation between HMGA1 and SAC genes expression was detected in human colon carcinomas indicating a novel mechanism by which HMGA1 contributes to cancer progression.

## INTRODUCTION

The High Mobility Group A (HMGA) protein family is comprised of three proteins: HMGA1a and HMGA1b, which are encoded by the *HMGA1* gene through alternative splicing [1], and the related HMGA2 protein, encoded by a distinct gene [2]. These proteins are nonhistone architectural nuclear factors, able to bind the minor groove of AT-rich DNA sequences through three “AT-hook” domains. The involvement of HMGA proteins in embryogenesis, cell proliferation, differentiation, apoptosis and, above all, cancer development has been extensively demonstrated [3]. In particular, HMGA proteins seem to play their major physiologic role during embryonic development where they are abundantly expressed, whereas their expression is low or negligible in normal adult tissues [4, 5]. Conversely, HMGA overexpression is a feature of malignant neoplasias [3], where it represents a poor prognostic index, as it often correlates with a reduced survival [6]. It has been previously demonstrated that HMGA proteins have oncogenic activity since their overexpression leads to the transformation of rat fibroblasts [7] and human epithelial breast cells [8], and transgenic mice overexpressing the HMGA proteins develop multiple neoplasias [9–13].

The mechanisms accounting for cell transformation induced by the HMGA proteins are essentially based on their ability to activate or inhibit the expression of genes involved in the control of cellular proliferation, invasion and apoptosis [3, 14–16].

A hallmark of human cancer is genome instability, whose prominent form is represented by chromosomal instability (CIN). This can consist in gain or loss of whole chromosomes, translocation/deletion/duplication of chromosome segments or polyploidy. Alterations in chromosome number or aneuploidy are found in nearly all major human tumor types [17], generally associated with the most aggressive forms of cancer. Alterations of the genes coding for proteins involved in Spindle Assembly Checkpoint (SAC), frequently observed in human malignant neoplasias, account for aneuploidy of cancer cells [18]. However, mutations in SAC genes are quite rare in human cancers (reviewed in [19]), whereas their deregulation is more frequent. Indeed, downregulation of *Bub1* expression has been detected in a subset of acute myeloid leukemia [20], whereas upregulation of *Bub1* levels has been reported in lymphomas [21], breast [22] and gastric cancers [23, 24]. Therefore, these studies demonstrate that both upregulation and downregulation of SAC genes can cause a checkpoint impairment leading to CIN.

Since HMGA expression is associated with a highly malignant phenotype that is characterized by CIN which accelerates tumor progression leading to a higher malignant state, the aim of this work has been to investigate whether HMGA1 proteins are able to regulate the expression of SAC genes, and, thereby, evaluate HMGA1 role in CIN.

Herein we report that HMGA1 increases the transcription of *Bub1, Bub1b, Mad2l1* and *Mps1/Ttk* genes involved in the SAC. We have found that HMGA1 knock-down compromises the mitotic checkpoint activity, and consistently HMGA1-depleted cells show a higher percentage of metaphases with unaligned chromosomes and a reduced prometaphase time compared to control cells, indicating a SAC impairment. Finally, human colon carcinomas show high SAC gene expression that correlates with HMGA1 protein levels.

## RESULTS

### HMGA1 increases the transcription of *Bub1, Bub1b, Mad2l1* and *Ttk* genes involved in the SAC

In order to identify a possible role of HMGA1 in the induction of chromosomal instability we have investigated, first, the ability of HMGA1 to regulate the expression of SAC genes, focusing our attention on *Bub1, Bub1b, Mad2l1* and *Ttk* genes.

These genes have been chosen because their promoters contain AT-rich regions, which represent putative binding sites for HMGA1 proteins, flanking the consensus binding sites for the same transcription factors (Supplementary Figure S1), some of which have been described as interactors of HMGA1 proteins (Supplementary Figure S1).

**Figure 1:**
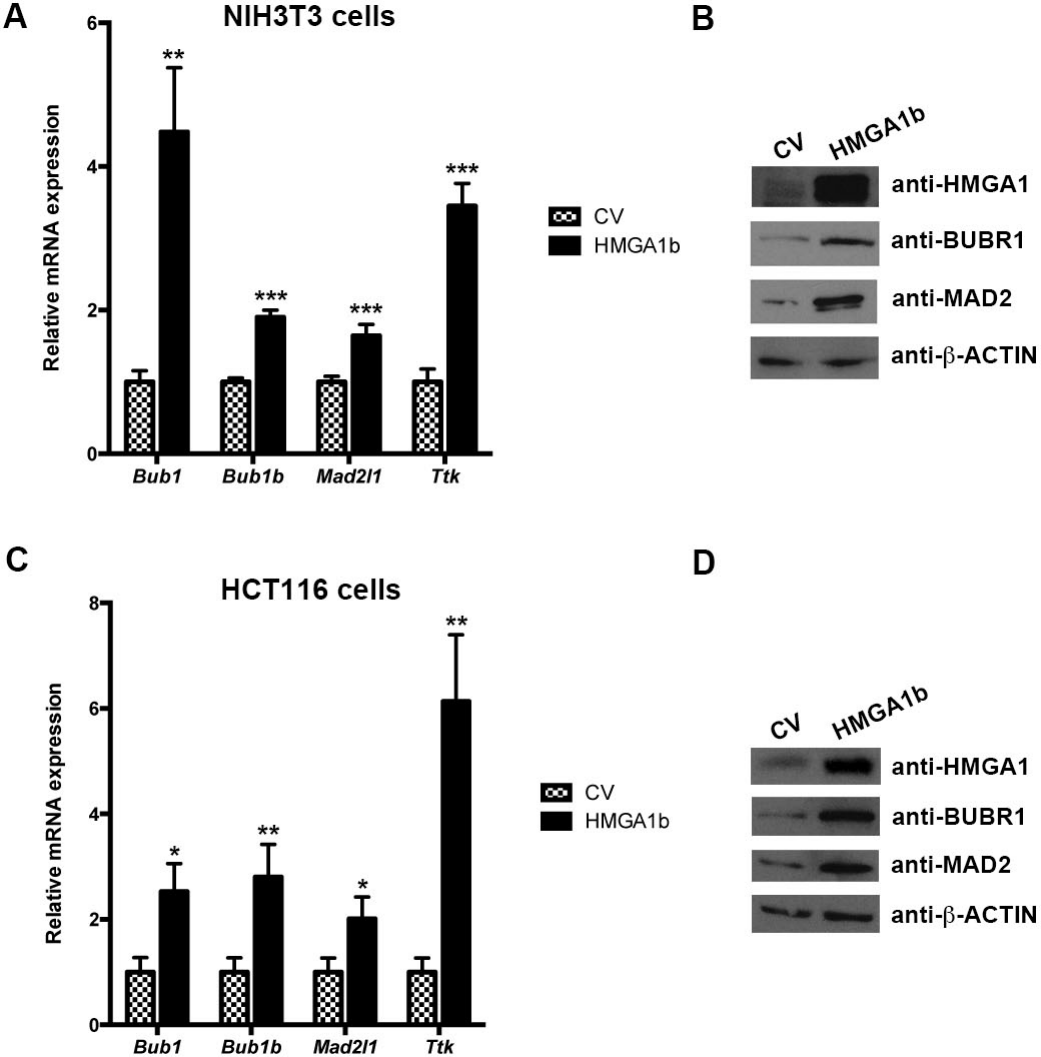
HMGA1 increases mitotic spindle regulator genes at both RNA and protein level RNA and proteins extracted from NIH3T3 cells transiently transfected with pCEFL-HA-*Hmga1b* carrying the *Hmga1b* cDNA or the backbone vector (CV) were analyzed by qPCR for *Bub1, Bub1b, Mad2l1* and *Ttk* expression **A.** and western blotting with the indicated antibodies **B.** The actin expression level has been used for normalization. Data are mean ± SD of a representative experiment performed in triplicate. *p < 0.05; **p < 0.01; ***p < 0.001, Student's *t* test. **C and D.** RNA and proteins extracted from HCT116 cells treated as in (A) were analyzed by qPCR for *Bub1, Bub1b, Mad2l1* and *Ttk* expression (C) and western blotting with the indicated antibodies (D) The actin expression level has been used for normalization.

Therefore, we have evaluated the expression of *Bub1, Bub1b, Mad2l1* and *Ttk* at mRNA level by quantitative PCR (qPCR) in mouse NIH3T3 cells transfected with the pCEFL-HA-*Hmga1b* expression vector encoding HMGA1b. As shown in Figure 1A, the expression of *Bub1, Bub1b, Mad2l*1 and *Ttk* genes is higher in the cells overexpressing HMGA1 in comparison with the empty vector-transfected NIH3T3 cells (CV). Parallel results were obtained when total cellular extracts from the same cells were analyzed by western blotting with the antibodies raised against BUBR1 and MAD2 proteins (Figure 1B). Moreover, the same results have been obtained in human colorectal HCT116, SW620 and SW48 cells overexpressing or not the HMGA1b protein (Figure 1C and 1D, and Supplementary Figure S2).

These results suggest a role of the HMGA1b protein in the activation of SAC gene expression.

### HMGA1 binds *Bub1, Bub1b, Mad2l1* and *Ttk* promoters *in vivo*

**Figure 2:**
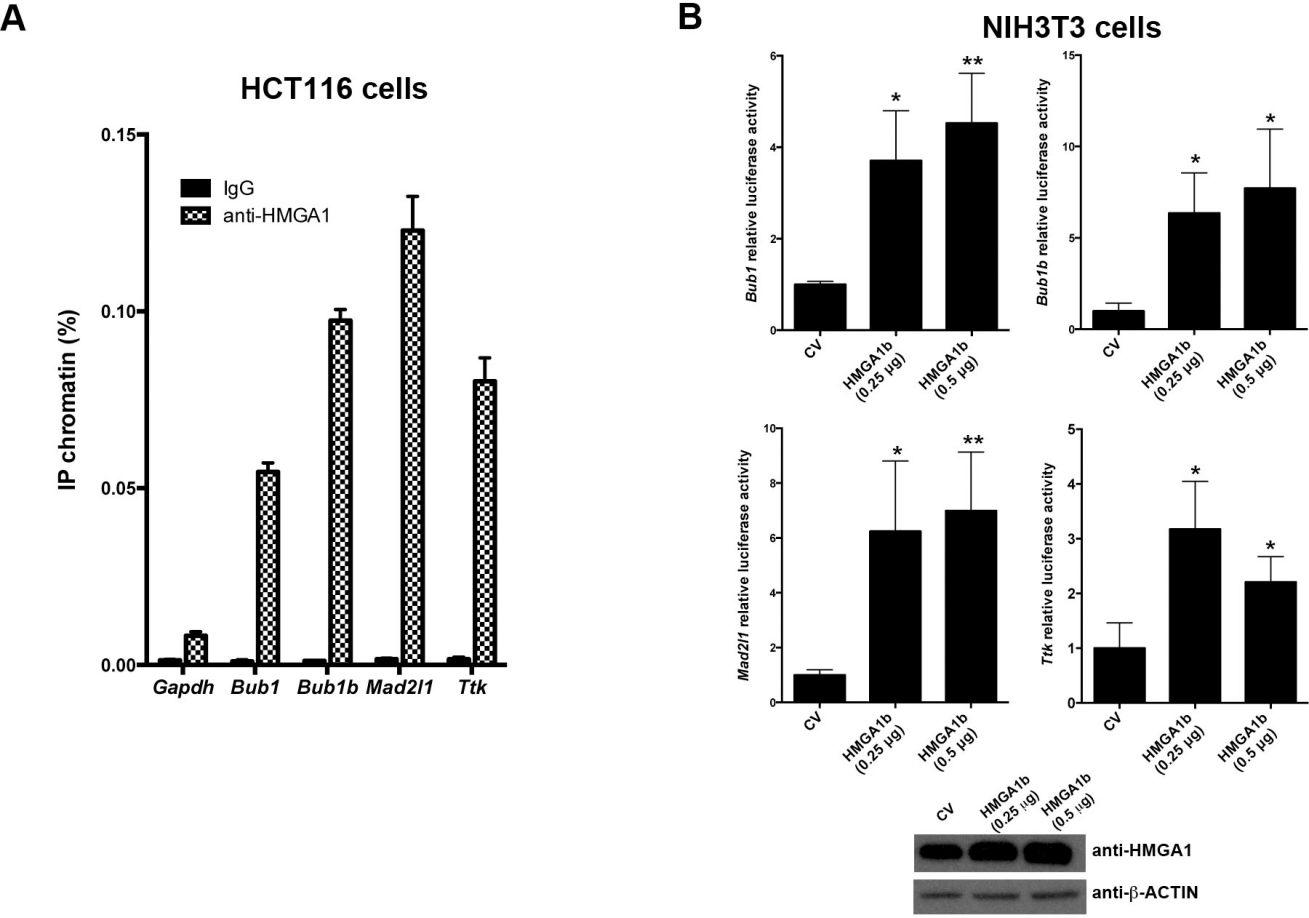
HMGA1 protein binds *Bub1, Bub1b, Mad2l1 and Ttk* promoters *in vivo* and increases their transcriptional activity **A.** Soluble chromatin from HCT116 cells has been immunoprecipitated with anti-HMGA1 antibodies. Then, the DNAs have been amplified by qPCR using primers covering different regions of *Bub1, Bub1b, Mad2l1* and *Ttk* promoters. *GAPDH* promoter and nonspecific IgG instead of anti-HMGA1 were used as control of the specificity of the HMGA1 binding to the indicated promoters. Data are mean ± SD of a representative experiment performed in triplicate. See also Supplementary Figure S1. **B.** Analysis of *Bub1, Bub1b, Mad2l1* and *Ttk* luciferase reporter activities in NIH3T3 cells transiently transfected with empty vector (CV), or 0.25 and 0.5 μg of pcDNA3.1-*Hmga1b* expression vector. All the transfections were performed in duplicate. Data are mean ± SD of three independent experiments. *p < 0.05; **p < 0.01, Student's *t* test. Western blotting analysis of HMGA1 protein from one representative experiment was shown (bottom). Actin was used to equalize protein loading.

As described above*, Bub1, Bub1b, Mad2l1 and Ttk* promoter regions contain some AT-rich DNA sequences which are the preferred binding sites for HMGA1 proteins. Therefore, we analyzed the ability of the HMGA1 proteins to bind the promoter regions of these genes by chromatin immunoprecipitation (ChIP) assays. Thus, HCT116 cells were cross-linked with formaldehyde, and DNA-chromatin complexes were subjected to immunoprecipitation with anti-HMGA1 or anti-IgG antibodies used as control. The recovered DNA was subsequently analyzed by qPCR, using primers spanning specific AT-rich regions of the indicated promoters. Specifically, the following AT-rich regions of these promoters: −760/−567 region of the *Bub1* promoter, −740/−550 region of the *Bub1b* promoter, −442/−198 region of the *Mad2l1* promoter, and −380/−194 region of the *Ttk* promoter, containing putative binding sites for HMGA1 proteins (see Supplementary Figure S1), were analyzed. The immunoprecipitated DNA has also been amplified using primers for the glyceraldehyde 3-phosphate dehydrogenase (Gapdh) gene promoter as negative control. As shown in Figure 2A, occupancy of *Bub1, Bub1b, Mad2l1* and *Ttk* promoters by HMGA1 has been detected in the anti-HMGA1-precipitated chromatin from HCT116 cells. Conversely, no amplification was observed with anti-IgG precipitates and when primers for the *Gapdh* promoter were used, indicating the specificity of the binding of HMGA1 proteins to the *Bub1, Bub1b, Mad2l1* and *Ttk* promoters.

Finally, to assess the functional consequences of the HMGA1 binding to these promoters, we investigated whether HMGA1 proteins were able to regulate these promoters performing luciferase activity assays in NIH3T3 cells. To this aim, NIH3T3 cells have been co-transfected with a reporter vector (pGL3-Basic) carrying the firefly-luciferase gene under the control of *Bub1, Bub1b, Mad2l1* and *Ttk* promoters, with different amounts of pcDNA3.1-*Hmga1b* and renilla-luciferase reporter vector (used for normalization). Insert-less pcDNA3.1 vector has been used as control (CV). As shown in Figure 2B, HMGA1 increases *Bub1, Bub1b, Mad2l1* and *Ttk* promoter activities.

Taken together, these results demonstrate that HMGA1 is able to bind to the *Bub1, Bub1b, Mad2l1* and *Ttk* promoters, and increase their transcriptional activity strongly supporting a critical role of HMGA1 in the regulation of SAC gene expression.

### HMGA1 depletion impairs the mitotic checkpoint activity

**Figure 3:**
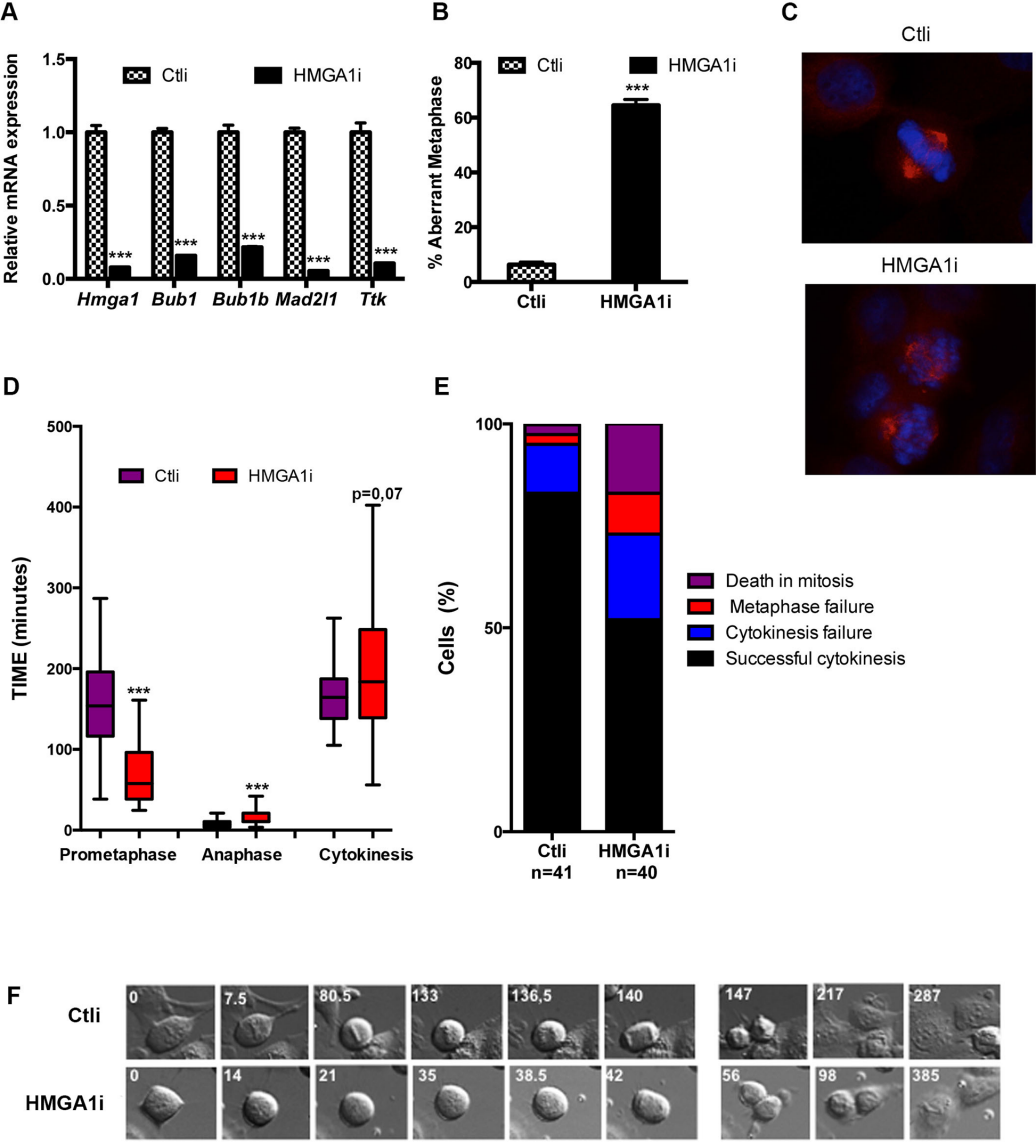
HMGA1 depletion impairs the mitotic checkpoint **A.** Control (Ctli) and HMGA1-depleted (HMGA1i) HeLa cells were tested for the expression of HMGA1 and SAC genes by qPCR. **B.** HeLa cells were transfected with Ctli and HMGAi siRNA as in A, fixed 48 hrs post transfection and stained with DAPI (blue) and anti-β-tubulin Ab (red) to identify the DNA and the mitotic spindle, respectively. About 150 metaphases per sample were scored for the presence of aligned chromosome. The data are represented as mean ± SD. Differences between Ctli and HMGA1i are statistically significant. ***p < 0.001, Student's *t*-test. **C.** Representative immunostainings are shown (right). **D.** HeLa cells treated as in A and analyzed 48 h post transfection by time lapse video. For each case, duration of the different phases of the mitosis and of the cytokinesis was measured. The duration of the indicated mitotic phases is reported in box plot graph (Whisker diagram). ***p < 0.001, Student's *t*-test. **E.** The percentage of cells with the indicated outcome is reported. **F.** Still images related to Supplementary Movies S1 and S2. See also Supplementary Figure S3.

Since the above described results indicate that HMGA1 is able to activate the expression of SAC genes acting at transcriptional level, we investigated whether HMGA1-induced SAC genes deregulation affects the mitotic checkpoint in mammalian cultured cells. To this aim, we used HeLa cells that have a functional SAC and are often used as a model for studying the checkpoint function. Since HeLa cells express HMGA1 protein at high levels, the further over-expression of HMGA1 failed to induce a consistent up-regulation of SAC genes. For this reason, considering that both up-regulation and down-regulation of one or more SAC genes impair the checkpoint, we evaluated whether HMGA1-silencing by RNAi is able to down-regulate SAC genes expression in HeLa cells. To this aim we transfected HeLa cells with siRNA targeting the *HMGA1* gene (HMGA1i cells) or with control siRNA (Ctli cells). As expected, we observed a drastic downregulation of SAC gene expression (*Bub1, Bub1b, Mad2l1* and *Ttk*) at mRNA (Figure 3A) and protein level (Supplementary Figure S3). The analysis of mitotic cells by immunofluorescence showed the presence of a high number of unaligned chromosomes in metaphase in HMGA1i cells but not in the scrambled oligonucleotide transfected HeLa cells (Figure 3B and 3C). Subsequently, to evaluate the role of HMGA1 in the mitotic checkpoint regulation, we used live-cell microscopy to follow mitotic progression in individual cells. Filming of mitoses in HeLa HMGA1i and Ctli cells revealed several mitotic errors in HMGA1i cells. The most prominent phenotype was a reduction of pro-metaphase time, i.e. from the round-up to the chromosome segregation, in HMGA1i cells (*t* = 70.21 ± 40.35 min (*n* = 32) *versus* 154.52 ± 61.46 min in Ctli (*n* = 32); Figure 3D and 3F and Supplementary Movies S1 and S2). We have observed that HMGA1i cells exhibited also an anaphase delay (*t* = 14.7 ± 8.89 min (*n* = 39) *versus* 8.05 ± 5.02 min in Ctli (*n* = 23); Figure 3D and 3F and Supplementary Movies S1 and S2) and that they showed a slightly higher cytokinesis time (from the early telophase to abscission; *t* = 199.5 ± 83.65 min *n* = 30) than that of Ctli cells (*t* = 161.28 ± 38.43 min *n* = 24), associated to a higher percentage of cytokinesis failure resulting in binucleated cells (21% versus 12% in Ctli cells; Figure 3D and 3E). Moreover, we observed a higher percentage of death in mitosis and of metaphase failure in the HMGA1i than in Ctli cells (Figure 3E).

Altogether, these results suggest that the deregulation of SAC genes induced by HMGA1 depletion impairs the activity of the checkpoint.

### HMGA1 expression levels directly correlate with the expression of genes involved in SAC

**Figure 4:**
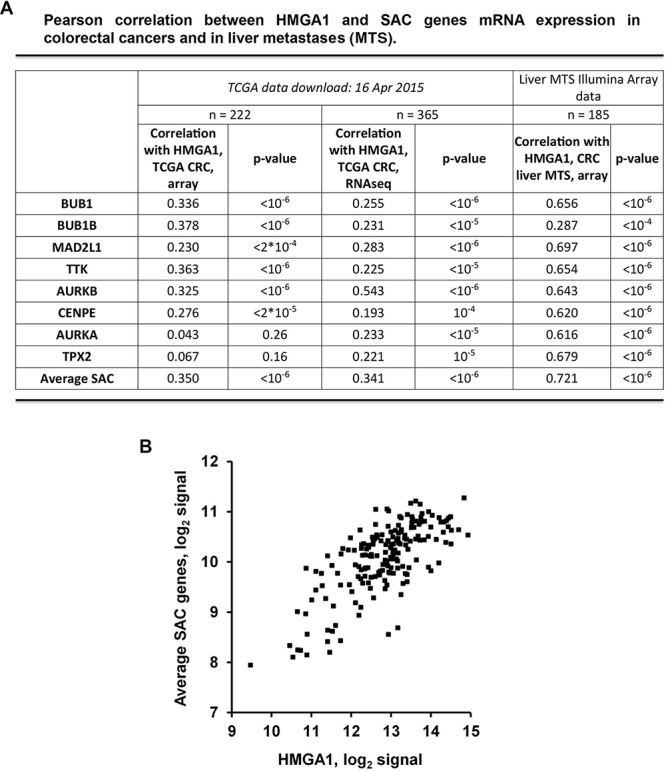
HMGA1 and SAC genes expression correlates in colorectal cancer samples and in liver metastases **A.** Correlation between the expression of *HMGA1* and of the SAC genes *BUB1, BUB1B, MAD2L1, TTK, AURKB, CENPE, AURKA* and *TPX2* in global mRNA profiles of colorectal cancer and liver metastases (MTS). 365 and 222 clorectal carcinoma samples were profiled by The Cancer Genome Atlas (TCGA; http://cancergenome.nih.gov/) with, respectively, RNAseq and DNA microarrays. 185 liver MTS samples were profiled with Illumina array. **B.** Dot-plot depicting the correlation between *HMGA1* and average SAC genes in liver MTS.

We evaluated the correlation between the expression of *HMGA1* and of the SAC genes *BUB1, BUB1B, MAD2L1* and *TTK*, in global mRNA profiles of colorectal cancer generated by The Cancer Genome Atlas (TCGA; http://cancergenome.nih.gov/) for 365 and 222 samples profiled with, respectively, RNAseq and DNA microarrays. *HMGA1* was found to be significantly correlated with each of the SAC genes both in RNAseq and microarray data (Pearson r between 0.22 and 0.38; *p* < 10^−6^ in 6 comparisons, and < 2*10^−4^ in the remaining two) (Figure 4A). We extended the analysis also to other SAC genes (*AURKB, CENPE, AURKA* and *TPX2)* obtaining the same results for two of them. Interestingly, the correlation further raised when the expression of the four SAC genes was averaged (r = 0.34 in RNAseq and 0.35 in array) (Figure 4A and 4B).

It is noteworthy that a stronger correlation between HMGA1 and SAC gene mRNA expression was found when liver metastases from colon carcinomas (*n* = 185) were analyzed by Illumina array (Figure 4A). In particular, we obtained a Pearson *r* > 0.62 and a *p* value < 10^−6^ for seven of the eight analyzed SAC genes, with a further increase for the averaged SAC genes expression (Pearson *r* = 0.72; *p* < 10^−6^).

**Figure 5:**
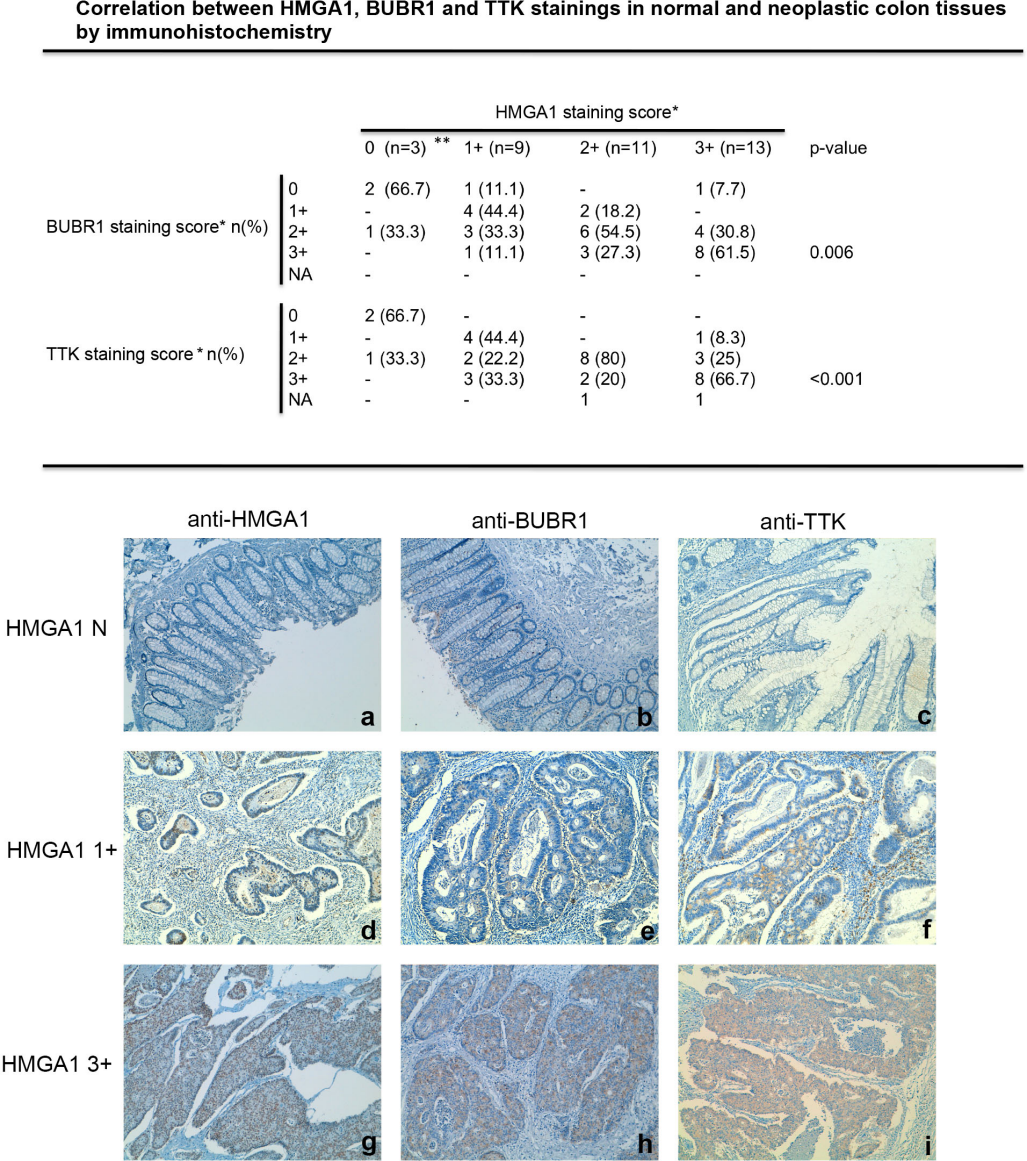
HMGA1 proteins expression correlates with BUBR1 and TTK expression in colorectal cancer samples Correlation between HMGA1, BUBR1 and TTK stainings in normal and neoplastic colon tissues by immunohistochemistry. *HMGA1, BUBR1 and TTK staining scores were: negative (N), <10% of positive cells; +, positive cells; 1+, 10–40% of positive cells; 2+, 41–70% of positive cells; 3+, 71–100% of positive cells. n, number of cases; %, percentage of examined cases; NA, not assessable; **normal control tissues. The correlation between HMGA1 and BUBR1 and between HMGA1 and TTK immunoreactivity are statistically significant: *p* = 0.006 and *p* < 0.001, respectively, calcuted by χ^2^ test. Representative images of paraffin sections analyzed by immunohistochemistry using anti-HMGA1, anti-BUBR1 and anti-TTK antibodies are shown. Immunostaining of normal mucosa negative (N) for HMGA1 **a.** BUBR1 **b.** and TTK **c.** are shown. Immunostaining of a well-differentiated colon carcinoma showing a weak immunoreactivity for HMGA1 **d.** BUBR1 **e.** and TTK **f.** are shown. Immunostaining of moderately differentiated colon carcinoma showing a strong immunoreactivity for HMGA1 **g.** BUBR1 **h.** and TTK **i.** are shown. Magnification is 10X.

Therefore, we attempted to confirm the association of HMGA1 expression with that of two SAC genes (BUBR1 and TTK) at protein level by immunohistochemical analyses of paraffin-embedded tissues evaluating the expression of these proteins in 36 colon tissue specimens (three normal colon tissues and 33 colon carcinoma). The results of this analysis are summarized in Figure 5. Indeed, as previously reported [25, 26], HMGA1 expression was abundant in the colon carcinoma samples, whereas it was not detectable in normal colonic mucosa. Interestingly, a weak immunoreactivity was observed in normal colon mucosa after staining with antibodies raised *versus* BUBR1 and TTK, whereas colon carcinoma samples showed a strong immunoreactivity that significantly correlated with HMGA1 staining (*p* = 0.006 for BUBR1 and *p* < 0.001 for TTK). In fact, 55.5% and 44.4% of tumor samples with a weak HMGA1 staining (1+) had negative or weak staining also for BUBR1 and TTK respectively, whereas 61.5% and 66.7% of tumor samples with a strong HMGA1 immunoreactivity (3+) had also a strong immunoreactivity for BUBR1 and TTK, respectively. Representative cases of normal colon mucosa (a, b, c), colon carcinoma samples with a weak HMGA1 immunoreactivity (d, e, f) and colon carcinoma samples with a strong HMGA1 reactivity (g, h, i) stained with antibodies versus BUBR1 and TTK are shown in Figure 5.

Therefore, human colon carcinoma show SAC gene overexpression that strongly correlates with that of HMGA1, likely contributing to cancer progression.

## DISCUSSION

Overexpression of HMGA proteins, a general feature of human malignancies [3], has a critical role in cancer progression since it inhibits p53-dependent apoptosis by modulating transcription of p53 [27] and p53-target genes (reviewed in [14]), impairs DNA repair [28] and induces the epithelial-mesenchymal transition (EMT) [29, 8]. Consistently, HMGA protein expression is associated with a highly malignant phenotype being a marker of poor prognosis for cancer patients as their overexpression correlates with the resistance to anti-cancer therapies [28] and a reduced survival [6].

Also alterations in chromosome number or aneuploidy are a feature of highly malignant neoplasias and it is due to defects in the mitotic checkpoint that controls chromosome segregation during mitosis [17]. Alterations of the spindle assembly checkpoint genes levels likely account for aneuploidy in several human cancer (reviewed in [30, 31]) since mitotic checkpoint impairment and aneuploidy in human tumour cells are often associated with changes in the SAC gene-encoded protein levels [30]. It has also been reported that in some tumour cells these changes occur through altered transcriptional regulation by tumour suppressors or oncogene products [32, 33].

Therefore, in order to investigate the mechanisms accounting for the association of HMGA1 overexpression and chromosome instability, we have analyzed the ability of the HMGA1 proteins to transcriptionally regulate the genes involved in SAC.

Here, we report that HMGA1 proteins are able to activate the expression of the SAC genes *Ttk, Mad2l1, Bub1* and *Bub1b* binding to their promoter regions. Consistently, HMGA1i-interfered HeLa cells (HMGA1i) show downregulation of these genes, whereas HMGA1-overexpression in several human colon cancer and NIH3T3 cells induces their upregulation. Moreover, the analysis of mitotic cells by immunofluorescence showed the presence of a high number of unaligned chromosomes in metaphase in HMGA1i cells associated to a reduction of prometaphase time. This alteration suggests that the HMGA1i cells start the anaphase without waiting for the proper chromosome alignment due to SAC impairment. Interestingly, the HMGA1-interfered HeLa cells resemble the cells in which the SAC gene *Bub1b* was depleted [34], indicating that HMGA1 depletion affects the mitotic checkpoint in cultured cells.

Notably, we have found a strong correlation between HMGA1 and BUBR1 and TTK protein levels in human colon carcinomas (Figure 4). Moreover, analysis of the TCGA expression data generated on CRC showed a statistically significant correlation, with the highest Pearson index of 0.54, between HMGA1 and SAC genes expression, evaluated either by microarray analysis or by RNAseq. this correlation strongly increased in liver metastases from colon carcinomas (pearson r = 0.72), supporting the idea that HMGA1-induced SAC genes deregulation may be important during tumour progression.

Altogether, these findings suggest that SAC gene overexpression induced by HMGA1 may have a role in colorectal cancer progression causing chromosome instability. Indeed, although it has also been demonstrated that mice carrying conditional *Bub1* mutation develop severe defects ranging from early lethality to tumorigenesis [35], even SAC gene overexpression has been reported to impair the spindle assembly checkpoint playing a critical role in cancer progression. In fact, MAD2 overexpression is a common event in many human tumors [36, 37], and its overexpression increases nondisjunction events and aneuploidy in human fibroblasts and cell lines [32]. Moreover, *Bub1* and *Bub1b* expression levels are drastically upregulated in gastric cancer associated with tumor cell proliferation [23]. Furthermore, colorectal cancer patients showing *Bub1* and *Bub1b* upregulation have a shorter relapse-free survival with respect to the groups showing normal expression of these genes [38].

In conclusion, based on the results presented here, we propose a novel mechanism by which HMGA1 overexpression contributes to cancer progression activating SAC gene expression, thereby inducing chromosomal instability that eventually leads to a more advanced cancer status.

## MATERIALS AND METHODS

### Cell cultures, transfections and plasmids

NIH3T3 and HeLa cell lines were cultured in DMEM with 10% FBS, L-glutamine, and antibiotics (Invitrogen, Carlsbad, CA). HCT116, SW620 and SW48 were cultured in McCoy's with 10% FBS, L-glutamine, and antibiotics (Invitrogen). For live-cell imaging, cells were cultured in DMEM medium without phenol red, supplemented with heat-inactivated FBS (Invitrogen). HCT116, SW620, SW48 and NIH3T3 cells were transfected using Lipofectamine-Plus reagent (Invitrogen) according to manufacturer's instructions. The pcDNA3.1-*Hmga1b* and pCEFL-HA-*Hmga1b* vectors were previously described, respectively [39, 40]. The pLuc-*Bub1b* reporter plasmid and pT81-*Mad2l1* promoters were gifts of Dr. P. Carbon [41] and Dr. CW Lee [42], respectively. The *Bub1* promoter region was amplified by PCR from human genomic DNA using the following primers:

lucBub1Fw: 5′-AATTCTCGAGGCTTGAAGCTGT TTGACAGG-3′
lucbub1Re: 5′-AATTAAGCTTCACATTCCAAAC CCAGGAAG-3′

The forward and reverse primers contain, respectively, the recognition sites for the restriction enzymes *Hind* III and *Xho*I. The amplified fragment was cloned into pGL3-Basic Firefly luciferase vector (Promega, Fitchburg, WI, USA) at the *Xho*I and *Hind* III sites upstream the luciferase gene. Human *Ttk* promoter spanning from −524 to +72 relative to the transcription start was previously reported [43].

### Luciferase assay

Cells were plated in 6 wells plates and after 24 hours were transfected with 100 ng of pLuc-*Bub1*, pLuc-*Bub1b*, pLuc-*Mad2l1* or pLuc-*Ttk* expression vectors together with 50 ng of pCMV-Renilla plasmid and with different amount of pcDNA3.1-*Hmga1b* expression vector. Luciferase and Renilla activities were assessed with the Dual-Light Luciferase system (Promega), 48 h after the transfection. Luciferase activity was normalized for the Renilla activity. All the experiments were performed three times in duplicate and the mean ± SD was reported.

### Protein extraction, western blotting, and antibodies

Cells were lysed in lysis buffer containing 1% NP40, 1 mM EDTA, 50 mM Tris-HCl (pH 7.5) and 150 mM NaCl, supplemented with complete protease inhibitors mixture (Roche Branford, CT, USA). Total proteins were separated by SDS-polyacrylamide gel electrophoresis and transferred to nitrocellulose membranes (Amersham, Rainham, UK) by elettroblotting. Membranes were blocked with 5% non-fat dry milk and incubated with antibodies anti-actin (sc-1616, Santa Cruz Biotechnology), anti-HMGA1 [44], anti-BUBR1 (612503, BD Transduction Laboratories), anti-TTK (C-19, sc-540 Santa Cruz Biotechnology), anti-MAD2 (610678, Transduction Laboratories).

### Chromatin immunoprecipitation (ChIP) assay

48 hours after transfection, cells have been treated with formaldehyde 1%, washed and then lysed isolating the nuclei. Then the nuclei have been in turn lysed and chromatin has been sonicated. Chromatin has been immunoprecipitated using anti-HMGA1 antibody [39] or normal rabbit IgG as negative control. After the immunoprecipitation, the chromatin has been incubated overnight at 65°C with DNAse-free RNAse (Roche). Next day, the samples have been incubated 3 hours at 50°C with 0, 5% SDS and 0, 5 mg/ml Proteinase K (Roche) and after the DNA has been purified with a phenol-chloroform extraction. For qPCR analysis, 2 μl out of 150 μl immunoprecipitated DNA was used with primers described below. *Gapdh* promoter amplicon was used as negative control in all the experiments. Input DNA was used as positive control.

Primers used were:

Bub1-prom −760/−567 Fw 5′-AACCCATCACTTTC CAGTGC-3′
Bub1-prom −760/−567 Re 5′-ACATCCCAGATGCTG AAACC-3′
Bub1b-prom −740/−550 Fw 5′-GCAAGAAGAAGAC CCTGTCTC-3′
Bub1b-prom −740/−550 Re 5′-ATGCTATGGTTCCC AAGGTG-3′
Mad2l1-prom −442/−198 Fw 5′-ACCTTATTCCTGT CCTGCCC-3′
Mad2l1-prom −442/−198 Re 5′-CCACAGCTTTACA GGGTTCG-3′
Ttk-prom −380/−194 Fw 5′-CCGCAAACAGATCAA CGAG-3′
Ttk-prom −380/−194 Re 5′-CGTGAGAGCCCTTCTC AATC-3′
Gapdh-prom-Fw 5′-CCCAAAGTCCTCCTGTTT CA-3′
Gapdh-prom-Re 5′-GTCTTGAGGCCTGAGCTA CG-3′

### RNA interference, RNA extraction and quantitative PCR

RNA interference was obtained by HMGA1-specific siRNA sequences (QiagenHs_HMGA1_5 (SI02662023) Sense strand GGACAAGGCUAACAUCCCATT and Antisense strand UGGGAUGUUAGCCUUGUCCAG] using Lipofectamine RNAi MAX (Invitrogen), according to manufacturer's instructions. Qiagen AllStars control siRNA (SI03650318) was used as negative control. Cells were transduced with 40 nM of siRNA using RNAiMAX reagent (Invitrogen) according to the manufacturer's instructions and analyzed after 48 hours. Red fluorescent oligonucleotides (Block-it, Invitrogen) were used to evaluate transfection efficiency. In our conditions, about 80% of the cells transfected by siRNA molecules. Total RNA was isolated as already described [45]. qPCR analysis for *Bub1, Bub1b, Mad2l1, Ttk* and *Hmga1* was performed using the Power SYBR Green PCR Master Mix (Applied Biosystems) according to manufacters's instructions with following primer sequences:

humanHMGA1-Fw 5′-CAACTCCAGGAAGGAAA CCA-3′
humanHMGA1-Re 5′-AGGACTCCTGCGAGAT GC-3′
humanBub1-Fw 5′-ACACCATTCCACAAGCTT CC-3′
humanBub1-Re 5′-CGCCTGGGTACACTGTTT TG-3′
humanBub1b-Fw 5′-TGGAAGAGACTGCACGAC AG-3′
humanBub1b-Re 5′-CAGGCTTTCTGGTGCTTA GG-3′
humanTtk-Fw 5′-ACCAAGCAGCAATACCTTG G-3′
humanTtk-Re 5′-ACTGACAAGCAGGTGGAA AG-3′
humanMad2l1-Fw 5′-GACATTTCTGCCACTGTT GG-3′
humanMad2l1-Re 5′-AACTGTGGTCCCGACTCT TC-3′
humanActin-Fw 5′-CCAACCGCGAGAAGATGA-3′
humanActin-Re 5′-CCAGAGGCGTACAGGGAT AG-3′
mouseBub1-Fw 5′-CAAGGACCTTCCTGCTTC TG-3′
mouseBub1-Re5′-GACTTGGACCCCTCAATTCC-3′
mouseBub1b-Fw5′-GCCAGATTGCAGATTGCTT C-3′
mouseBub1b-Re 5′-GGACAGATGGAACAGGAC AG-3′
mouseTtk-Fw 5′-ATATGGCCCCAGAAGCAAT C-3′
mouseTtk-Re 5′-CCCCAAGGACCAGACATCA C-3′
mouseMad2l1-Fw-5′-AGAAACTGGTGGTGGTC ATC-3′
mouseMad2l1-Re 5′-CGAACACCTTCCTCTTTT GC-3′
mouseActin-Fw 5′-CTAAGGCCAACCGTGAAA AG-3′
mouseActin-Re 5′-ACCAGAGGCATACAGGGA CA-3′

To calculate the relative expression levels we used the 2-ΔΔCT method [46]. Primers specific for the actin were used for normalization of qPCR data.

### Statistical analysis

Student's *t* test was used to determine the significance for all the quantitative experiments. Error bars represent the standard deviation (SD) of the average. A *p*-value < 0.05 was considered statistically significant.

The χ^2^ test was used to test the relationship between the immunoreactivity of HMGA1 and that of BUBR1 and TTK in colon samples. The test indicates how much of the association is accounted for linear trend. Statistical significance for all the tests was assessed by calculating the *p*-value.

For correlation analysis, TCGA expression data for HMGA1, BUB1, BUB1B, MAD2L1 and TTK in colorectal cancer were downloaded as z-scores from the cBioPortal (http://www.cbioportal.org). Pearson correlation between HMGA1 and SAC gene expression was calculated with Microsoft Excel. Statistical significance of the observed values was assessed on the online portal “Simple Interactive Statistical Analysis” (http://www.quantitativeskills.com/sisa/statistics/correl.htm).

### Immunofluorescence

Cells plated on cover-slides in 12 wells plates were fixed in 4% formaldehyde in PBS and permeabilized in a solution of 0.25% Triton X-100 in PBS. Anti β-tubulin antibody conjugated to CY3 (Sigma) was used to identify mitotic spindle in mitotic cells, and DAPI staining was used to identify chromosomes. Cells were observed with a fluorescent microscope (Zeiss, magnification 63X or 100X).

### Live-cell imaging

Cells were seeded in slides 8-well (80826, ibiTreat, Ibidi) and observed under an Eclipse Ti inverted microscope (Nikon) using a 40x objective (Nikon). During the observation, cells were kept in a microscope stage incubator (Basic WJ, Okolab) at 37°C and 5% CO2. DIC images were acquired over a 24 hr period by using a DS-Qi1Mc camera. Image and video processing were performed with NIS-Elements AR 3.22.

### Immunohistochemistry

Immunohistochemical analysis of paraffin-embedded tissues was performed with antibodies against HMGA1, BUBR1 and TTK, using polyclonal antibodies above mentioned, as previously described [47]. No staining was observed when normal colon samples were stained without the primary antibodies (data not shown).

## SUPPLEMENTARY FIGURES AND MOVIES



## Abbreviations

Mad2l1: mitotic arrest deficient-like 1
Bub1: budding uninhibited by benzimidazoles 1
CIN: chromosome instability
SAC: spindle assembly checkpoint
HMGA1: high mobility group A1
RNAi: RNA interference
