# Supplementary material for: Deregulation of HMGA1 expression induces chromosome instability through regulation of spindle assembly checkpoint genes
Source: Oncotarget. 2015 May 15;6(19):17342–53. doi: 10.18632/oncotarget.3944 (PMC4627312; doi:10.18632/oncotarget.3944)
Supplement: Supplementary file 1 [file oncotarget-06-17342-s001.pdf]

SUPPLEMENTARY FIGURES AND MOVIES

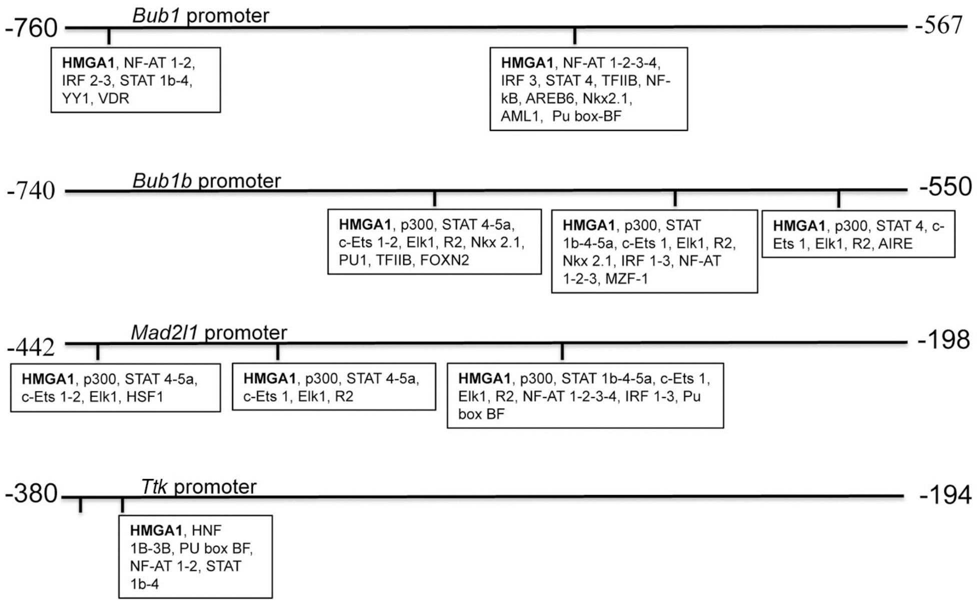

Supplementary Figure S1: Schematic representation of putative binding sites for HMGA1 proteins and for other transcription factors on the *Bub1, Bub1b, Mad2l1* and *Ttk* promoter regions analyzed by ChIP analysis (http://alggen.lsi.upc.es/cgibin/promo_v3/promo/promoinit.cgi?dirDB=TF_8.3).

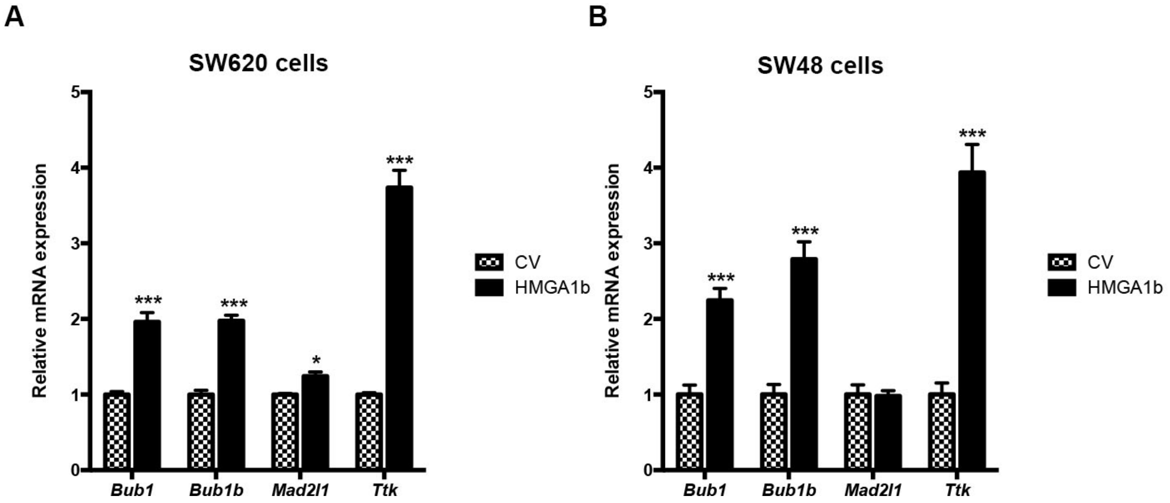

Supplementary Figure S2: HMGA1 increases SAC genes expression in colon cancer cells. RNA extracted from SW620 **A.** and SW48 **B.** cells transiently transfected with pcDNA3.1-*Hmga1b* carrying the *Hmga1b* cDNA or the backbone vector (CV) was analyzed by qPCR for *Bub1, Bub1b, Mad2l1* and Ttk expression. The actin expression level has been used for normalization. Data are mean ± SD of a representative experiment performed in triplicate. *$p < 0.05$; ***$p < 0.001$, Student's *t* test.

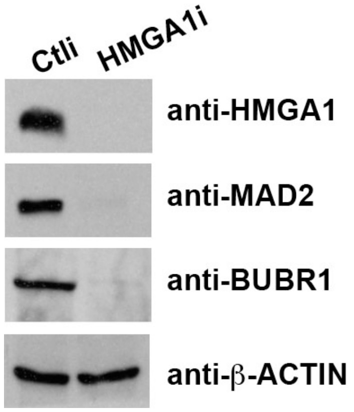

Supplementary Figure S3: Depletion of HMGA1 expression in HeLa cells reduces SAC genes expression. Control (Ctli) and HMGA1-depleted (HMGA1i) HeLa cells were tested for the expression of HMGA1 and SAC proteins by Western blotting with the indicated antibodies. Actin was used for normalization.

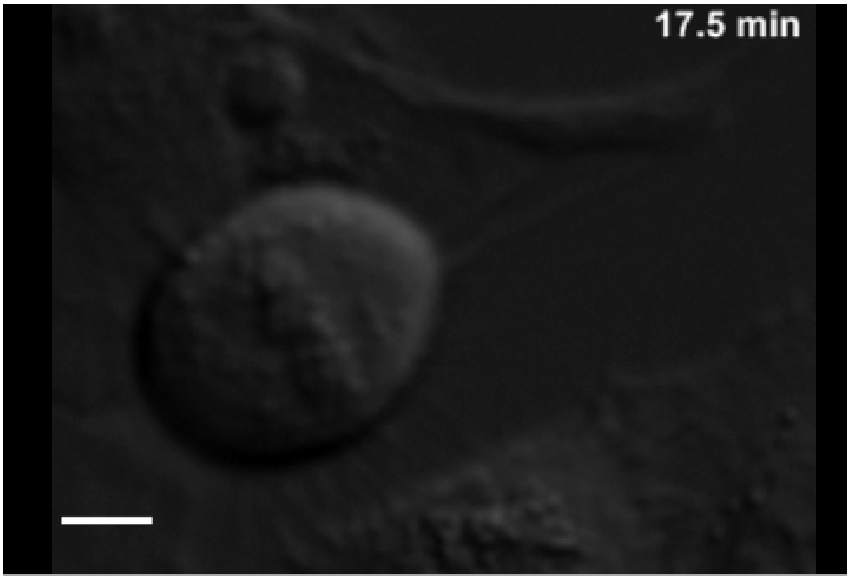

Supplementary Movie S1: Time-Lapse Movie of asynchronous, proliferating HeLa-Ctli cells. Time-lapse movie of asynchronous HeLa Ctli cells related to Figure 3. Phase contrast images of cells were captured every 3.5 min. The display rate is one frame every 250 millisecond. Still images of this video are shown in Figure 3F (upper panel).

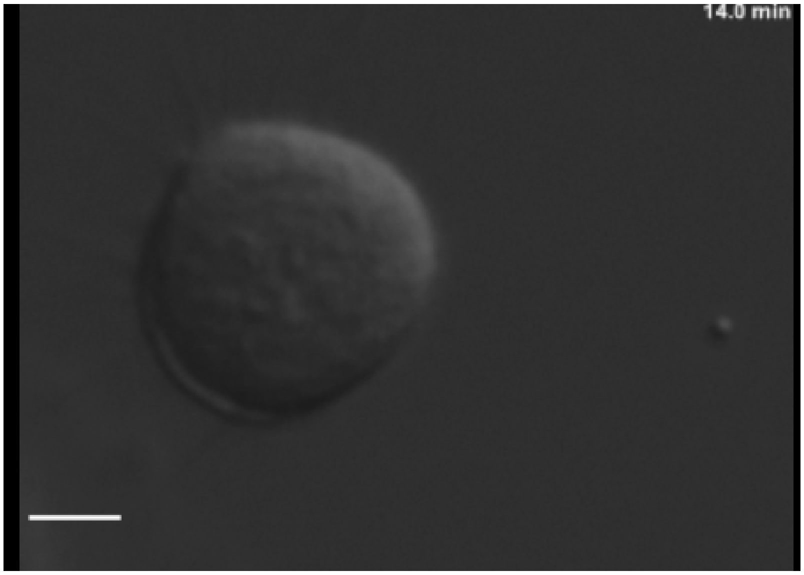

Supplementary Movie S2: Time-Lapse Movie of asynchronous, proliferating HeLa-HMGA1i cells. Time-lapse movie of 3 asynchronous HeLa HMGA1i cells Related to Figure 3. Phase contrast images of cells were captured every 3.5 min. The display rate is one frame every 250 millisecond. Still images of this video are shown in Figure 3F (lower panel).
